# Neue Therapiemöglichkeiten beim metastasierten HER2-low-Mammakarzinom: Konsequenzen für die histopathologische Diagnostik

**DOI:** 10.1007/s00292-022-01124-x

**Published:** 2022-10-13

**Authors:** Carsten Denkert, Annette Lebeau, Hans Ulrich Schildhaus, Christian Jackisch, Josef Rüschoff

**Affiliations:** 1 Institut für Pathologie, Philipps-Universität Marburg und Universitätsklinikum Marburg (UKGM), Baldingerstr. 1, 35043 Marburg, Deutschland; 2 Institut für Pathologie, Universitätsklinikum Hamburg-Eppendorf (UKE), Hamburg, Deutschland; 3 Gemeinschaftspraxis für Pathologie, Lübeck, Deutschland; 4 Discovery Life Sciences, Kassel, Deutschland; 5 Pathologie Nordhessen, Kassel, Deutschland; 6 Klinik für Gynäkologie und Geburtshilfe, Sana Klinikum Offenbach GmbH, Offenbach, Deutschland

**Keywords:** HER2-Gene, Immunkonjugate, Immunhistochemie, Zielgerichtete molekulare Therapie, Trastuzumab, HER2 genes, Immunoconjugates, Immunohistochemistry, Molecular targeted therapy, Trastuzumab

## Abstract

Die standardisierte HER2-Bestimmung beim Mammakarzinom und bei anderen Tumoren ist eine wichtige Aufgabe der Pathologie. Ziel der bisherigen Bestimmung war es, zuverlässig diejenigen Tumoren zu identifizieren, die eine Überexpression des HER2-Proteins aufweisen, die in der Regel mit einer Genamplifikation einhergeht. Nur in dieser Gruppe von Tumoren war eine zielgerichtete Anti-HER2-Therapie sinnvoll und erfolgversprechend. Durch neue Substanzen und die Ergebnisse klinischer Studien beim metastasierten Mammakarzinom hat sich dies nun geändert. Es konnte gezeigt werden, dass Trastuzumab-Deruxtecan, ein Konjugat aus einem Anti-HER2-Antikörper und einer zytotoxischen Substanz, auch dann einen Anti-Tumor-Effekt aufweist, wenn nur eine geringe Expression von HER2 im Tumorgewebe vorliegt.

Die aktuellen Daten bedeuten einen Paradigmenwechsel für die Behandlung von Patientinnen, deren Tumoren bislang als HER2-negativ eingeordnet wurden. Ziel ist es jetzt, neben den Tumoren mit einer HER2-Überexpression (IHC 3+) auch die Tumoren mit einer geringen HER2-Expression (HER2-low, definiert als IHC 1+ oder 2+/ISH-negativ) zuverlässig zu identifizieren. Aufgrund der therapeutischen Konsequenzen ist es wichtig, die diagnostischen Algorithmen und Befundtexte in allen pathologischen Instituten sehr kurzfristig an die neuen Erfordernisse anzupassen. Unabhängig davon ergeben sich neue wissenschaftliche Fragen und Herausforderungen für die Standardisierung, die aktuell bearbeitet werden.

## Abstract (en)

The overexpression of HER2 in breast cancer is a classic example for molecular targeted therapy, and it has been shown that classical anti-HER2 therapeutics were only effective in patients with HER2 overexpressing tumors. Therefore, in recent decades, pathologists have been focused on the reliable identification of HER2 overexpressing tumors. Based on the results of recent clinical trials in metastatic breast cancer with antibody-drug conjugates (ADCs), this diagnostic strategy for evaluation of HER2 is currently changing. It has been shown that the ADC trastuzumab-deruxtecan is effective not only against tumors with classical HER2 overexpression, but also against HER2-low tumors. These clinical trial results lead to a paradigm shift in the treatment of patients whose tumours were previously classified as HER2 negative. In addition to the identification of HER2 (score 3+) overexpressing tumors, it is necessary to identify HER2-low expressing tumors (defined as an immunohistochemistry (IHC) score of 1+ or IHC2+ with negative in situ hybridization).

Due to the therapeutic consequences, it is important to quickly adapt the diagnostic workup and reporting to the new requirements. In addition, the new therapeutic options for anti-HER2 therapy lead to new challenges for standardization as well as to new scientific questions for the characterization of tumors with low HER2 expression.

Basierend auf den Ergebnissen klinischer Therapiestudien [18] wurde Trastuzumab im Jahr 2000 in der EU als Therapieoption für das HER2-positive metastasierte Mammakarzinom zugelassen, 2006 erfolgte die Zulassung in der adjuvanten Situation. In der Folge wurden weitere Anti-HER2 Therapiestrategien etabliert, darunter die duale Blockade mit Trastuzumab/Pertuzumab und die Therapie mit dem Antikörper-Wirkstoff-Konjugat (ADC, „antibody drug conjugate“) T‑DM1. Die therapeutischen Möglichkeiten reichten somit von der Monotherapie mit Trastuzumab bis zur dualen horizontalen oder vertikalen Blockade mit Anti-HER2 gerichteten Antikörpern oder Tyrosinkinaseinhibitoren wie Lapatinib oder Neratinib.

Therapieprinzip bei allen bisherigen Therapien war, dass die Therapie nur bei HER2-positiven Tumoren wirksam war, die eine starke Überexpression auf Proteinebene und/oder eine Genamplifikation aufwiesen. In der NSABP-B47-Studie [9] und in einer translationalen Untersuchung an der GeparQuattro-Studie [3] konnte bestätigt werden, dass bei Tumoren ohne HER2-Überexpression kein therapeutischer Nutzen von Trastuzumab besteht. Für die Pathologie bestand damit der klare Untersuchungsauftrag einer zuverlässigen Identifikation derjenigen Tumoren, die eine HER2-Überexpression aufweisen. Die Bestimmung folgte den Vorgaben, die zunächst im Rahmen der Zulassung von Trastuzumab durch die FDA [6] und danach von der ASCO/CAP in den HER2 Guidelines 2007 [32], 2013 [33] und 2018 [34], formuliert wurden. Parallel wurden regelmäßige Ringversuche etabliert.

Unter Berücksichtigung neuer Studienergebnisse, die auf dem ASCO 2022 und zeitgleich im *New England Journal of Medicine* [14] vorgestellt wurden, ergibt sich ganz aktuell die Notwendigkeit, auch die Tumoren mit niedriger HER2-Expression (HER2-low, definiert als 1+ oder 2+ mit negativer ISH) zuverlässig zu identifizieren und dies im histologischen Bericht zu dokumentieren. Eine Änderung der Auswertekategorien ist dabei zunächst gar nicht notwendig, allerdings sollten im Einzelfall die Teststrategien und die Formulierung im histologischen Bericht angepasst werden, um für klinische Entscheidungen eine optimale Grundlage zu bieten.

## Neue therapeutische Optionen – Ergebnisse aktueller klinischer Studien

Trastuzumab-Deruxtecan (DS-8201a; T‑DXd) ist ein neuartiges, gegen HER2 gerichtetes Antikörper-Wirkstoff-Konjugat (ADC) [19]. Die ADC-Technologie besteht darin, spezifische Antikörper durch eine chemische Verbindung (Linker) mit einem Toxin zu verbinden. Die gegen HER2 gerichteten Antikörper transportieren das Toxin spezifisch zur Tumorzelle, wo das Toxin – in diesem Fall ein Topoisomeraseinhibitor – abgespalten und internalisiert wird, um die zytotoxische Wirkung zu entfalten. Damit sind die systemischen Auswirkungen minimiert und Nebenwirkungen können vermieden werden. Trastuzumab-Deruxtecan ist nicht nur direkt in stark HER2-positiven Tumorzellen wirksam, sondern entfaltet seine Wirkung auch bei Karzinomzellen mit HER2-low-Expression. Aufgrund der Freisetzung des Toxins im Tumorgewebe kommt es zusätzlich zu einer Wirkung auf benachbarte Zellen (sog. Bystander-Killing-Effekt). Eine Übersicht über ausgewählte aktuelle klinische Studien zu Trastuzumab-Deruxtecan ist in Tab. 1 gezeigt.

**Table 1:** 

Studie	Tumortyp	Studiendesign	Ergebnis	Referenz
*Ausgewählte Studien beim Mammakarzinom*				
DESTINY-Breast01 Phase 2	HER2-positives metastasiertes Mammakarzinom	Phase 2 Trastuzumab-Deruxtecan nach Vortherapie mit T‑DM1	61 % Ansprechen	Modi et al., NEJM 2020 [15]
DESTINY-Breast03	Metastasiertes HER2-positives Mammakarzinom	Trastuzumab-Deruxtecan vs. T‑DM1	Studienziel erreicht PFS (progression-free survival/progressionsfreies Überleben): 75,8 % vs. 34,1 % Gesamtansprechrate 79,7 % vs. 34,2 %	Cortes et al., NEJM 2022 [2]
DESTINY-Breast04	Metastasiertes HER2-low-Mammakarzinom (IHC1+ oder 2+/SISHneg)	Trastuzumab-Deruxtecan (T-DXd) vs. Chemotherapie nach Wahl des Prüfarztes (Capecitabin, Eribulin, Gemcitabin, Paclitaxel)	Studienziel erreicht (Details siehe Text) *Es ergeben sich unmittelbare Konsequenzen für die HER2-Diagnostik*	Modi et al., NEJM 2022 [14]
Daisy-Studie	Metastasiertes Mammakarzinom (HER2-positiv, HER2-low und HER2-negativ)	Phase 2 Trastuzumab-Deruxtecan	Ansprechraten: HER2 3+: 71 % HER2-low: 37,5 % HER2-neg: 30,0 %	ESMO breast 2022 [16]
*Ausgewählte Studien bei anderen Tumoren*				
DESTINY-Gastric01	Fortgeschrittenes Karzinom des Magens oder des gastroösophagealen Übergangs mit klassischer Definition der HER2-Positivität, (3+ bzw. 2+/FISH positiv)	Phase 2 Trastuzumab-Deruxtecan (T-DXd) vs Chemotherapie	Die objektive Ansprechrate (ORR) und das Überleben (OS) waren dabei signifikant besser als im Chemotherapie-Kontrollarm (OS: 12,4 vs. 8,4 Monate)	Shitara et al., NEJM 2020 [26]
DESTINY-CRC-01	Kolorektales Karzinom ohne *RAS/BRAF*-Mutationen, klassisch HER2-positiv und HER2-low-positiv	Phase 2 Trastuzumab-Deruxtecan	HER2 IHC3+ bzw. IHC2+/ISH-positive Tumoren: objektives Therapieansprechen 45 % (sonstige Ergebnisse stehen noch aus)	Siena et al. Lancet Oncol 2021 [27]
DESTINY-Lung01	Nichtkleinzelliges Lungenkarzinom Aktivierende *ERBB2*-Mutation (unabhängig von HER2-Expression und *ERBB2*-Amplifikation)	Phase 2 Trastuzumab-Deruxtecan	Ansprechen in 55 %	Li et al., NEJM 2022 [12]

Für die aktuelle erweiterte klinische Indikation und die pathologische Diagnostik ist die beim ASCO 2022 vorgestellte DESTINY-Breast04-Studie [14] am relevantesten. In dieser Studie erhielten Patientinnen mit metastasierten HER2-low-Mammakarzinom, die schon eine oder zwei Linien einer Chemotherapie erhalten hatten, im Verhältnis 2:1 randomisiert entweder Trastuzumab-Deruxtecan (T-DXd) oder eine Chemotherapie nach Wahl des Prüfarztes (Capecitabin, Eribulin, Gemcitabin, Paclitaxel).

Das mediane progressionsfreie Überleben (PFS) in der HR+-Kohorte betrug in der T‑DXd-Gruppe 10,1 Monate. Das ist 4,7 Monate länger als in der Kontrollgruppe (5,4 Monate). Die Hazard Ratio (HR) für das PFS betrug 0,51 (95 % Konfidenzintervall [KI] 0,40–0,64; *p* < 0,0001). In der Gesamtpopulation ergab sich ein ähnlich signifikanter Vorteil mit einem medianen PFS von 9,9 Monaten in der T‑DXd-Kohorte und 5,1 Monaten in der Chemotherapiegruppe. Ein deutlicher Vorteil für T‑DXd zeigte sich auch für das Überleben (OS). In der HR+-Gruppe betrug das mediane OS mit T‑DXd 23,9 Monate, mit Chemotherapie 17,5 Monate. In der Gesamtkohorte waren die Ergebnisse zum medianen OS ähnlich vorteilhaft für das Antikörper-Wirkstoff-Konzentrat (T-DXd 23,4 Monate, Chemotherapie 16,8 Monate, HR 0,64; 95 % KI 0,49–0,84; *p* = 0,0010). Die Daten der Studie waren Grundlage für die Zulassung von Trastuzumab-Deruxtecan durch die FDA Anfang August 2022 [7].

Damit steigt der Anteil der Patientinnen mit metastasiertem Mammakarzinom, die von einer Anti-HER2-Therapie profitieren können: Zu den Patientinnen mit klassisch stark HER2-positiven Karzinomen (ca. 15 %; [15]) kommen diejenigen Patientinnen, bei denen eine HER2-low-Expression vorliegt (je nach Studienkohorte ca. 50 %) [29].

## Prinzipien des HER2-low-Scorings

Es sollte betont werden, dass seit den Zulassungsstudien für Trastuzumab Ende der 1990er-Jahre eine Einteilung der HER2-Expression in 4 Gruppen (0, 1+, 2+, 3+) besteht. Die positiven Studienergebnisse mit T‑DXd beim HER2-low-Mammakarzinom in der DESTINY-Breast04-Studie wurden auf Basis der aktuell gültigen ASCO/CAP-Empfehlungen 2018 [34] zur HER2-Bestimmung beim Mammakarzinom erzielt. Somit ist das Regelwerk auch für die HER2-low-Diagnostik bereits verfügbar.

Aufgrund der neuen Datenlage ergibt sich nunmehr eine klinisch relevante Dreiteilung der HER2-Expression, die im histologischen Befundbericht eindeutig übermittelt werden sollte: HER2-negativ (oder HER2-zero; IHC0), HER2-low (IHC1+ oder 2+/ISHneg) und HER2-positiv (IHC3+ oder 2+/ISHpos). Da sich aktuell nur für das Mammakarzinom Therapieoptionen ergeben, sollte die Bezeichnung HER2-low aktuell auch nur bei diesem Tumortyp verwendet werden.

**Figure 1:**
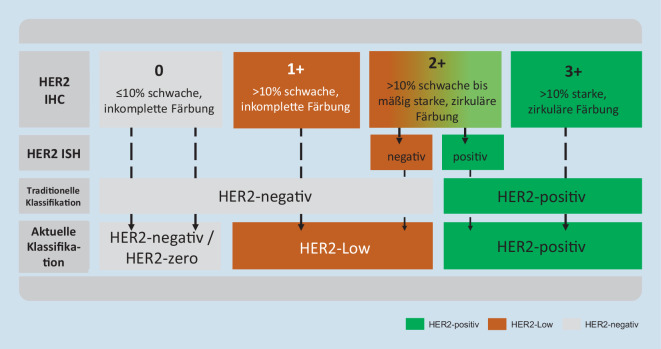

Basierend auf aktuell laufenden Studien zur Analyse der Interobservervarianz bei der HER2-low-Diagnostik wird folgende Vorgehensweise bei der Beurteilung der immunhistochemischen Färbung empfohlen (Abb. 1 und 2):

**Figure 2:**
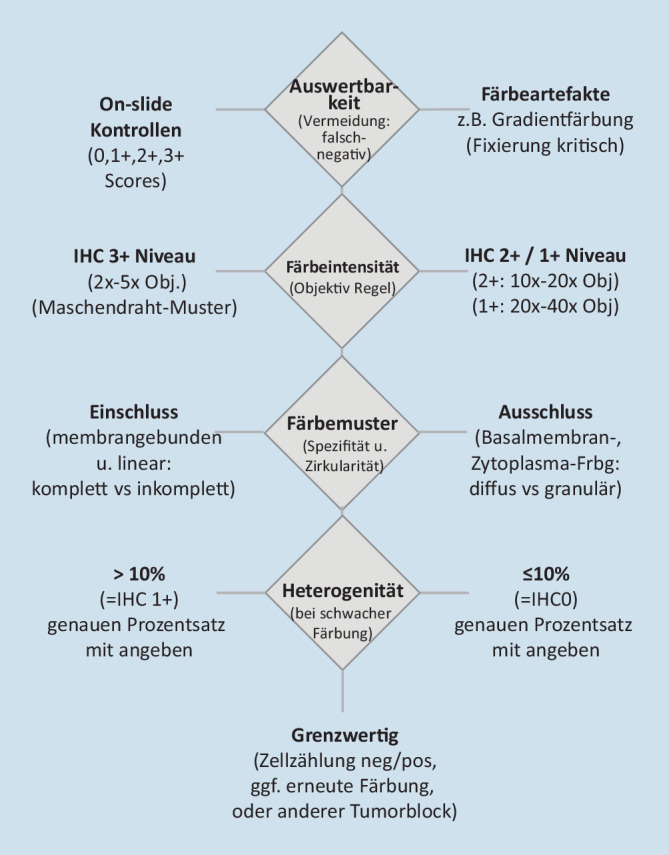

### Schritt 1: Anwendung der „Objektiv- oder Vergrößerungsregel“ [23]

**Figure 3:**
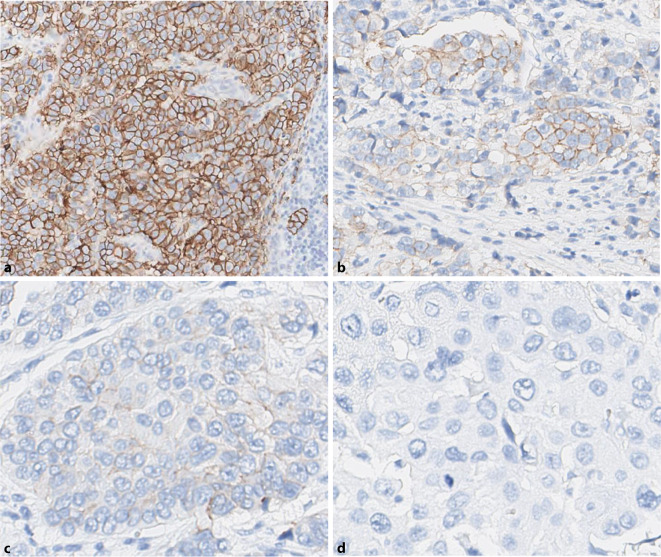

Hier sollte die gesamte Tumorfläche durchgemustert werden (Abb. 3). Entscheidend ist, bei welcher Vergrößerung eine membranäre HER2-Expression zu erkennen ist.

- Eine starke HER2-Färbung (IHC3+) ist schon bei Verwendung eines 2fach- oder 5fach-Objektivs als klare Membranfärbung zu erkennen.
- Eine mittelstarke HER2(IHC2+)-Färbung ist typischerweise erst im 10fach- oder 20fach-Objektiv als lineare Membranfärbung an den Zell-Zell-Kontaktstellen zu erkennen.
- Eine schwache (IHC1+)Färbung ist in der Regel erst mit dem 40fach-Objektiv eindeutig als Membranfärbung zu identifizieren.

Die Verwendung dieses einfachen Auswerteschemas ermöglicht als ersten Schritt eine semiquantitative Bestimmung der verschiedenen Färbeintensitäten und reduziert den Einfluss der Subjektivität.

### Schritt 2: Färbemuster – Zirkularität der Membranfärbung

Zur Klärung der Frage, ob es sich um einen IHC1+- oder IHC2+-Score handelt, ist im zweiten Schritt zusätzlich die Vollständigkeit der Membranfärbung (Zirkularität) zu bestimmen. Typischerweise ist bei IHC2+ eine vollständige, zirkuläre Färbung der Zellmembran nachweisbar, während bei IHC1+ lediglich eine partielle Färbung besteht. Eine Ausnahme kann bei drüsenbildenden Tumoren bestehen, bei denen der apikale Bereich bei der Färbung ausgespart sein kann (somit entsteht ein U‑förmiges Färbemuster, das als IHC2+ zu interpretieren ist).

**Figure 4:**
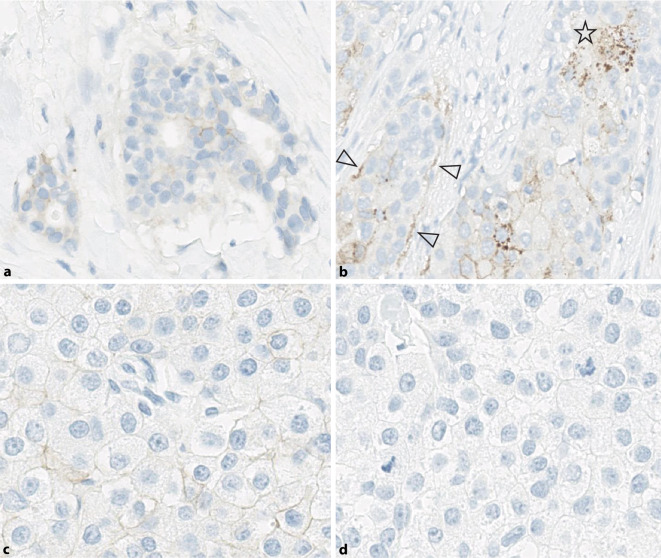

Zudem ist auf abweichende Färbemuster zu achten, z. B. zytoplasmatische Anfärbungen oder lineare Färbung lediglich im Basalmembranbereich. Diese Färbemuster sind nicht zu berücksichtigen (somit IHC0). Erste Analysen zur Interobservervariabilität zeigen, dass diese Färbemuster Hauptursache für eine zu hohe Bewertung sind (z. B. IHC 1+ statt 0, oder 2+ statt 1+; Abb. 4).

### Schritt 3: Prozentsatz der Tumorzellen mit HER2-Expression

Für einen IHC-Wert von 1+ (entsprechend HER2-low) müssen mindestens 10 % der Tumorzellen eine inkomplette Membranfärbung aufweisen, während für einen Score 2+ mindestens 10 % der Tumorzellen eine komplette, zirkuläre Membranfärbung aufweisen müssen. Somit ist als dritter Schritt der Prozentsatz der den diagnostischen Score definierenden Tumorzellen zu bestimmen. Es ist sinnvoll, diesen Prozentsatz auch im histologischen Bericht mit anzugeben. Typische Formulierungen im Befund könnten z. B. sein: „Nachweis einer schwachen, inkomplett membranären Färbung in 25 % der Tumorzellen, somit IHC1+.“ Aufgrund der häufig heterogenen Färbeverteilung vor allem an der Grenze zwischen IHC1+ und IHC0 besteht hier eine besondere Herausforderung mit dem Risiko sowohl einer falsch-negativen als auch (seltener) falsch-positiven Beurteilung.

Problematisch sind die Fälle mit einer schwachen, inkompletten Färbung, die knapp unterhalb der 10 % Grenze liegt. In der Nähe des Cut-offs wird gerade bei einzelzelligem Wachstumsmuster (lobulär, tubulo-lobulär) die Tumorzellzahl eher überschätzt und damit der IHC Score unterschätzt (Abb. 4). Im Zweifel sollte man in solchen Fällen blickfeldweise Tumorzellen auszählen (Gesamtzahl und positive Zellzahl) und ggf. eine Mitbefundung durch Kollegen/innen einholen. In Abhängigkeit von der diagnostischen Ausgangssituation kann eventuell auch eine Färbewiederholung am selben oder an einem zweiten Tumorblock (z. B. am nachfolgenden Resektat) in Erwägung gezogen werden.

Für die Tumoren mit einer schwachen Expression unterhalb des 10 % cutoffs wird von einigen Autoren auch der Begriff HER2 „ultralow“ benutzt [31], der allerdings für aktuelle klinische Entscheidungen nicht relevant ist und daher aktuell in Befundberichten nicht verwendet werden sollte.

## Praxis der HER2-low-Testung

### Probenmaterial

Als Untersuchungsmaterial ist Material geeignet, das in 10 %igem, neutral gepuffertem Formalin zwischen 6 und 72 h fixiert und in Paraffin eingebettet wurde (FFPE-Material) [32, 33]. Bei Nachweis von Metastasen wird generell – unabhängig vom primären HER2-Status – eine erneute Bestimmung des Rezeptorstatus empfohlen, weil es zu einer Änderung des HER2-Status im Rahmen der Tumorprogression kommen kann. In etwa 10 % ist eine Änderung des HER2-Status bezogen auf die „alten“ Kategorien zu erwarten, wobei zumeist ein Expressionsverlust beobachtet wird (alte Kategorien: HER2-positiv → HER2-negativ) [25]. Erste Untersuchungen zur Frage der Änderung der HER2-Expression innerhalb der primär HER2-negativen Karzinome (alte Kategorie), zeigen eine Diskordanz der HER2-Expression (HER2-low versus HER2-zero) in bis zu 32 % der Fälle mit einer Tendenz zur Zunahme der HER2-low-Expression in fortgeschrittenen Mammakarzinomen [13, 28]. Im Rahmen der DESTINY-Breast04-Studie wurden die HER2-Bestimmungen an aktuellen Tumorbiopsien und an archiviertem Material der Primärtumoren durchgeführt [14]. Falls eine histologische Untersuchung von Metastasengewebe nicht möglich ist, kann dementsprechend auch auf FFPE-Material des Primärtumors zurückgegriffen werden. Falls im Rahmen der Primärdiagnostik bereits eine qualitätsgesicherte HER2-Immunhistochemie durchgeführt wurde, empfiehlt sich eine Reevaluation der Schnitte unter besonderer Berücksichtigung der aktuellen Erkenntnisse zur HER2-low-Bewertung. Eine Analyse mehrerer Gewebeproben einer Tumorlokalisation erscheint derzeit nicht angezeigt, da die Mehrheit der HER2-low-Karzinome ersten Erkenntnissen nach ein homogenes Färbemuster zeigen [14].

### Antikörper, Geräteplattformen, spezielle Situation in der EU/in Deutschland

Die Identifikation eines HER2-low-Status setzt eine Testung mittels HER2-Immunhistochemie voraus, somit ist eine alleinige Testung mittels In-situ-Hybridisierung nicht möglich. Die Validität und Reproduzierbarkeit der HER2-Bestimmung lässt sich mit standardisierten Testkits leichter gewährleisten, weshalb die Verwendung solcher Testkits exakt nach Angaben des Herstellers unter Nutzung des entsprechenden Färbeautomaten empfohlen wird.

Im Rahmen der DESTINY-Breast04 wurde der VENTANA HER2/neu(4B5)-Assay (Roche, Basel, Schweiz) verwendet. In Europa ist die Medikamentenzulassung grundsätzlich nicht an bestimmte diagnostische Testassays gebunden, das heißt, es dürfen auch andere Testassays oder Primärantikörper als „Laboratory Developed Tests“ (LDT) verwendet werden. In den USA hat die FDA definierte Testsysteme zur klassischen HER2-Evaluation zugelassen [8]. Dieser Prozess läuft aktuell auch für die Bestimmung des HER2-low Status zur Indikationsstellung für eine Therapie mit Trastuzumab-Deruxtecan.

In Deutschland wird neben dem 4B5-Assay auf den VENTANA BenchMark-Färbeautomaten vor allem der polyklonale Kaninchen-Antikörper von DAKO/Agilent (Santa Clara, CA, USA) auf dem Autostainer als Testkit eingesetzt (HercepTest for Automated Link Platforms, Agilent). Bei einem Vergleich der beiden Testkits an 500 Mammakarzinomen lag die prozentuale Gesamtübereinstimmung bei 73,5 % (95 %-CI 69,1–77,0) [21]. Der Hauptgrund für die Diskrepanzen zwischen den beiden Assays war, dass die Tumoren mit dem VENTANA 4B5 tendenziell in höhere HER2-Kategorien eingestuft wurden als mit dem HercepTest.

Ob diese Ergebnisse von anderen Arbeitsgruppen reproduziert werden und auch für den neuen Testassay HercepTest^TM^ mAb pharmDx (Dako Omnis) oder LDTs mit monoklonalen Primärantikörpern wie CB11, SP3 oder EP3 gelten, wird aktuell evaluiert. In einer aktuellen Vergleichsstudie zeigte der neue HercepTest-Assay (GE001) eine gegenüber 4B5 tendenziell etwas höhere Einstufung der HER2-Kategorien mit einer Gesamtübereinstimmung von 83,7 % [22].

Die beobachteten Diskrepanzen verdeutlichen aber, dass die Qualität der eingesetzten immunhistochemischen Nachweisverfahren zukünftig nicht nur im Hinblick auf ihre korrekte Identifizierung von HER2-positiven Karzinomen, sondern auch von HER2-low-Karzinomen betrachtet und geprüft werden müssen. Hierzu empfiehlt sich die Verwendung von On-Slide-Kontrollen unter Verwendung von Zelllinien mit definierten HER2-Färbemustern im niedrigen Bereich sowie die regelmäßige Teilnahme an Ringversuchen, die beispielsweise von der „Qualitätssicherungs-Initiative Pathologie“ angeboten werden.

## T-DXd in weiteren Indikationen und bei *HER2*-Mutation

Das *ERBB2*-Gen codiert für eine Rezeptortyrosinkinase, deren Expression und Aktivierung bei zahlreichen menschlichen Karzinomen eine Rolle spielt. Die pathogene Aktivierung kann dabei durch eine Genamplifikation mit konsekutiver Überexpression, aber auch durch pathogene Mutationen erfolgen. Dies betrifft neben dem Mammakarzinom unter anderem auch molekulare Subgruppen des Rektumkarzinoms [30], des Magenkarzinoms [11], des cholangiozellulären Karzinoms [1] und des Lungenkarzinoms [20]. Insofern ist es folgerichtig, dass die Wirksamkeit von T‑DXd derzeit auch in weiteren Indikationen klinisch erforscht wird.

In der Phase-II-Studie DESTINY-Gastric01 [26] wurden Patienten mit HER2-positivem (3+ oder 2+/ISH+) fortgeschrittenem Karzinom des Magens oder des gastroösophagealen Übergangs ab der Zweitlinie behandelt. Die objektive Ansprechrate (ORR) und das Überleben (OS) waren dabei signifikant besser als im Chemotherapiekontrollarm (OS: 12,4 vs. 8,4 Monate).

Auch für das kolorektalen Karzinom gibt es bereits erste publizierte Studiendaten [27]. Behandelt wurden Tumoren ohne *RAS/BRAF*-Mutationen in 2 Kohorten, mit klassischer HER2-Überexpression/Amplifikation oder mit HER2-low-Ausprägung. Für die Kohorte mit HER2 IHC3+- bzw. IHC2+/ISH-positiven Tumoren wurde ein objektives Therapieansprechen von über 45 % berichtet. Weitere Studiendaten stehen noch aus. Bei Lungenkarzinomen mit HER2-Mutationen konnte ein partielles Ansprechen oder eine vollständige Tumorregression in über 50 % der Patienten beobachtet werden [12]. Interessanterweise trat der Effekt sowohl bei den typischen Kinasedomänemutationen (*ERBB2*-Exon 20) als auch bei Mutationen auf, die die extrazelluläre Domäne betreffen – und war zudem offenbar unabhängig vom HER2-Expressionsstatus und einer *ERBB2*-Genamplifikation.

## Aktuelle Herausforderungen und wissenschaftliche Fragestellungen

Untersuchungen an einer Kohorte von 2310 Patientinnen mit Mammakarzinom aus klinischen Studien der German Breast Group [4] zeigen klinische und biologische Unterschiede zwischen HER2 vollständig negativen (IHC0, HER2-zero) und HER2-low-Tumoren. HER2-low-Tumoren sind häufiger hormonrezeptorpositiv, sie haben eine geringere Ansprechrate auf neoadjuvante Chemotherapie sowohl in der Gesamtkohorte als auch in der HR-positiven Subkohorte und zeigen eine verbesserte Prognose nach neoadjuvanter Therapie in der HR-negativen Subgruppe, die nicht auf eine neoadjuvante Therapie angesprochen hat. Ähnliche Ergebnisse, insbesondere die positive Korrelation zwischen HER2-low-Status und einem positiven Hormonrezeptorstatus, zeigten sich auch in andere Untersuchungen [24].

Eine weitere offene Frage ist, wie niedrig der HER2-Status werden darf, damit die Trastuzumab-Deruxtecan-Therapie noch wirksam ist. In der DAISY-Studie [5] zeigten sich Anhaltspunkte für eine Wirkung von auch bei HER2-negativen (IHC0) Tumoren, die möglicherweise eine minimale HER2-Expression unterhalb des Detektionslevels aufwiesen. Es ist aber nicht klar, ob sich diese Befunde auch in einer prospektiven Phase-3-Studie bestätigen lassen, sodass aktuell keine Relevanz für die histologische Diagnostik und Therapieplanung besteht.

Ein wichtiger Schwerpunkt für die Standardisierung der Bestimmung von HER2 wird die Ausweitung der Ringversuche auf HER2-low-positive Tumoren sein. Aktuelle Daten aus klinischen Studien deuten darauf hin, dass hier noch Verbesserungsbedarf besteht. In einer aktuellen Untersuchung [10] fand sich nur eine 26 %ige Konkordanz unter 18 Pathologen bei der Bewertung als 0 oder 1+, allerdings wurden hier die teilnehmenden Pathologen für das eigentliche Studienziel verblindet, d. h. es wurde ihnen nicht mitgeteilt, dass die Konkordanz zwischen IHC0 und IHC1+ evaluiert werden würde. Somit ist bei der Interpretation zu berücksichtigen, dass es bisher klinisch nicht relevant war, zuverlässig zwischen IHC0 und IHC1 zu unterscheiden. Die beiden Gruppen wurden deshalb teilweise in histologischen Befunden als HER2-negativ zusammengefasst. Erweiterte Analysen zu den HER2-Expressionsmustern, unter anderem aus der DESTINY-breast04-Studie, sind aktuell in der Auswertungsphase.

Es ist zu erwarten, dass die Konkordanz deutlich ansteigen wird, wenn allgemein bekannt ist, dass die zuverlässige Unterscheidung von IHC0 und IHC1+ für die Patientinnen neue therapeutische Optionen ermöglicht. Ein ähnlicher Zusammenhang fand sich auch bei der Konkordanz der klassischen HER2-Bestimmung: In einer Analyse der HER2-Bestimmung in GBG-Studien über 12 Jahre [17] konnte gezeigt werden, dass seit 2011 eine 92 %ige Übereinstimmung zwischen der Bestimmung der HER2-Überexpression in der lokalen Pathologie und der zentralen Bestimmung in der Studienpathologie bestand. In den Jahren vor 2006 gab es hingegen mit 25–50 % diskrepanten Fällen deutlich höhere Diskrepanzen. Hieraus wird deutlich, dass die diagnostische Standardisierung und Konkordanz ansteigen, sobald es eine Behandlungsindikation gibt. Dies lässt sich bei der Bestimmung der HER2-3+-Gruppe sehr gut nachvollziehen, somit ist eine ähnliche Verbesserung der Konkordanz auch für die HER2-low-Gruppe zu erwarten.

## Fazit für die Praxis

Aus den aktuellen Studienergebnissen der DESTINY-breast04-Studie und den daraus resultierenden neuen Therapieoptionen ergeben sich für die praktische diagnostische Pathologie wichtige Schlussfolgerungen:

- Im histologischen Bericht sollte eine klare Unterscheidung zwischen HER2 IHC0 und IHC1+ getroffen werden. Die beiden Gruppen sollten nicht als „HER2-negative“ Gruppe zusammengefasst werden.
- Die neue Gruppe sollte im Befundbericht eindeutig gekennzeichnet sein. Wir empfehlen die Verwendung des internationalen Begriffes „HER2-low“, alternativ könnte die deutsche Bezeichnung „HER2 niedrig exprimierend“ verwendet werden.
- Dabei sollte die Bezeichnung „HER2-low“ aktuell nur beim Mammakarzinom verwendet werden, die Therapieoption ergibt sich derzeit nur für das metastasierte Mammakarzinom. Die standardisierte Evaluation mit Erhebung und Dokumentation der einzelnen IHC-Scores sollte aber bei allen Tumortypen erfolgen.
- Die histologische Diagnostik basiert unverändert auf den aktuellen ASCO/CAP-Leitlinien für die HER2-Diagnostik. Auch die Bewertung der stark HER2-positiven Tumoren mittels Immunhistochemie (bei IHC3+) oder die Kombination aus IHC und In-situ-Hybridisierung (bei IHC2+) bleiben identisch. Dabei kann die Unterscheidung zwischen 0 und 1+ nur mittels Immunhistochemie getroffen werden.
- Für die Auswertung der Immunhistochemie wird das in diesem Artikel beschriebene standardisierte Vorgehen (1. Objektivregel – 2. Färbemuster – 3. Prozentsatz) empfohlen. Dabei sollte der Prozentsatz positiver Zellen im Befundbericht mit angegeben werden. Dies ist für die Abgrenzung zwischen IHC0 (≤ 10 % schwach positive Zellen) und IHC1+ (> 10 % schwach positive Zellen) relevant. Auf diese Weise kann man direkt erkennen, welche Tumoren sich im Grenzbereich befinden.
- Für die Bestimmung kann der Primärtumor oder eine Rebiopsie der Metastase verwendet werden, wobei die Leitlinien bei Tumorprogression als Standard eine Rebiopsie mit erneuter Bestimmung des Rezeptorstatus (einschließlich HER2) empfehlen, falls dies klinisch möglich ist.
